# Volatile Organic Compounds (VOCs) Removal from Indoor Air by Heterostructures/Composites/Doped Photocatalysts: A Mini-Review

**DOI:** 10.3390/nano10101965

**Published:** 2020-10-03

**Authors:** Alexandru Enesca, Cristina Cazan

**Affiliations:** Product Design, Mechatronics and Environmental Department, Transilvania University of Brasov, Eroilor 29 Street, 35000 Brasov, Romania; c.vladuta@unitbv.ro

**Keywords:** photocatalysis, heterostructures, composites, indoor air, volatile organic compounds

## Abstract

The impact of volatile organic compounds (VOCs) on indoor air quality and, furthermore, on human health is still a subject of research investigations considering the large increase in forms of cancer and related diseases. VOCs can be 10 times higher in indoor air concentrations then that of the outdoors, as a consequence of emissions from electronics, building materials and consumer goods. Direct transformation of VOCs in mineralization products seems to be an alternative to reduce indoor air contaminants. The advantage of photocatalysis implementation in indoor air treatment is given by the absence of additional chemicals (such as H_2_O_2_) and waste. The present mini-review presents a comparative study on VOCs photocatalytic removal considering the photocatalyst composition, morphology and specific surface. The sheet-like morphology seems to provide a higher number of active sites which may contribute to oxidative reactions. The insertion of materials able to increase light absorbance or to mediate the charge carrier’s transport will have a beneficial impact on the overall photocatalytic efficiency. Additionally, surface chemistry must be considered when developing photocatalysts for certain gas pollutants in order to favor molecule absorbance in the interfacial region. An energy consumption perspective is given based on the light intensity and irradiation period.

## 1. Introduction

Keeping high standards of indoor air quality parameters is a challenge considering that 80% is the average proportion of time individuals spend inside confined spaces. Based on geographical position and economic development, indoor air quality can have worse parameters than outdoor air. There are various categories of indoor air pollutants: CO_x_, NO_x_, small particles, volatile organic compounds (VOCs), etc. [1,2,3]. Among them, VOCs can reach ten times higher concentrations indoors than outdoors, as a consequence of emissions from electronics, building materials and consumer goods [4,5,6].

One way to deal with this issue is preventing pollution by source, but the economic repercussions of this measure on industry and economic development have delayed the practical implementation [7,8]. Instead, additional measures have been considered for reducing the impact of VOCs on human health: increased ventilation or absorbance in filters. The implications of these actions are controversial since they transfer the pollutants from one phase/space to another [9,10,11]. 

Direct transformation of VOCs in mineralization products seems to be an alternative to reduce indoor air contaminants [12,13]. Several advanced oxidation processes (AOPs) with good prospects for developing air cleaning technologies were considered: UV/ozonation [14,15,16], H_2_O_2_/ozonation [17,18,19] or photocatalysis [20,21,22]. The advantage of photocatalysis implementation in indoor air treatment is given by the absence of additional chemicals (such as H_2_O_2_).

This mini-review explores the possibilities of using a photocatalysis process to eliminate VOCs that are dangerous to human health, such as: toluene, *N,N*-dimethylformamide, acetone, dimethyl fumarate, formaldehyde, benzene, ethylbenzene, o-xylene, acetaldehyde, acetylene, 2-ethyl-1-hexanol, *N*-decane, *N*-hexane, benzaldehyde and trichloroethylene. The influence of photocatalyst composition, morphology, specific surface and light spectra on the photocatalytic efficiency was evaluated. The energy consumption based on light intensity and irradiation period was compared with the amount of pollutant removal. The photocatalytic efficiency refers to partial or total pollutant degradation according to the information provided in the literature. Due to the arbitrary reported photocatalysis experimental parameters and the lack of standardization, the scientific literature is abundant with investigations based on various input data that are difficult to compare.

## 2. Photocatalysis Mechanisms for VOCs Removal

The morphological properties have an important influence on the photocatalytic efficiency and are considered as the main driving force for photocatalysis processes in the gas phase. The low mass transfer resistance encountered in gas phase photocatalysis can be useful to develop high surface area photocatalysts [23,24,25]. Another important prerequisite in gas phase photocatalysis, besides large specific surface area and adsorption capacity, is represented by the high photon conversion efficiency [26]. These are the reasons why researchers have worked on developing complex structures (heterostructures, composites, doped materials, etc.) able to convert extended UV–Vis (and even Infrared (IR)) spectra as well as to efficiently use the incident photons for the photogeneration of free charge carriers [27,28,29].

**Figure 1:**
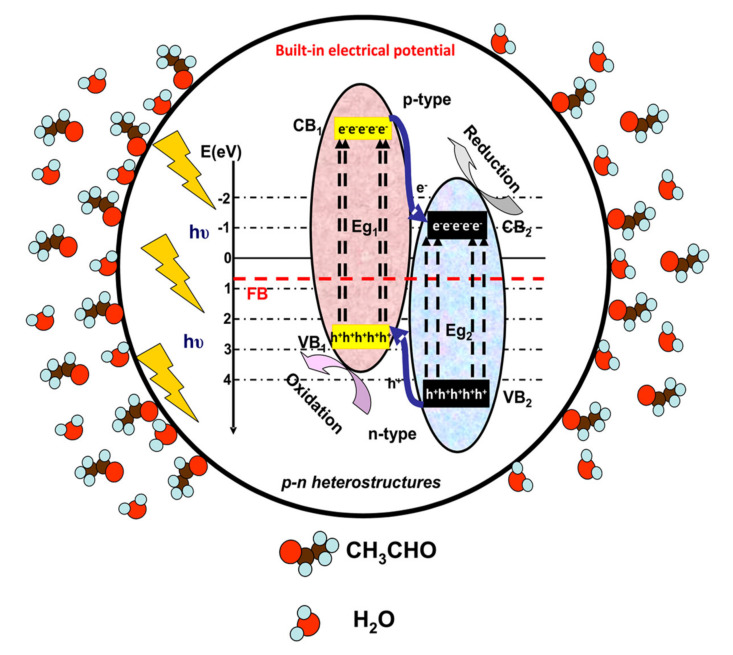
Charge carrier photogeneration during the initial irradiation period.

**Figure 2:**
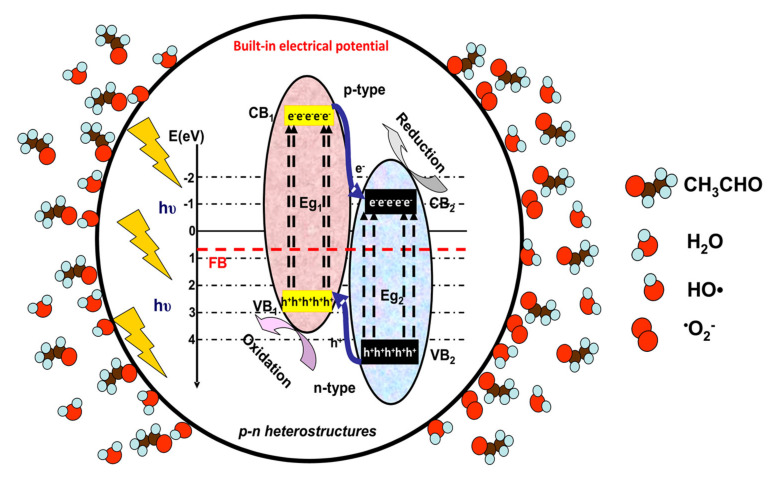
Development of oxidative and superoxidative species.

**Figure 3:**
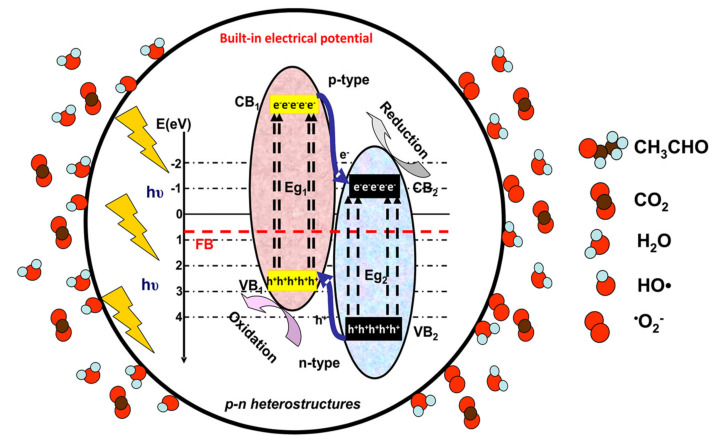
Photocatalytic degradation of acetaldehyde gas.

For pollutants in the gaseous phase, the net mass transfer from the bulk to the external surface of the photocatalyst has a significant impact on the overall efficiency and the rate of reaction. Figure 1, Figure 2 and Figure 3 illustrate the photocatalytic mechanism of acetaldehyde degradation using a p-n heterostructure. The photocatalytic degradation of VOCs can be induced by employing heterostructures or composites following type II, Schottky, Z-scheme or S-scheme mechanisms. Using multi-component photocatalysts is a suitable method to increase the charge carrier’s concentration and consequently the number of photogenerated oxidative species.

The development of inherent electric fields between the heterostructure partners induces a shift in individual Fermi levels, finally settling on a uniform value [30,31]. The gas molecules and the photocatalysts may the change function of the specific applications envisaged by researchers. The first step is represented by reaching the adsorption-desorption equilibrium in the absence of light irradiation. The duration depends on the surface chemistry between the gas molecules and catalysts as well as the catalyst porosity [32].

Under irradiation (step 2, Figure 1), the p-n heterostructure system generates charge carriers (e^−^ and h^+^) which move toward the surface and support (step 3, Figure 2) the formation of oxidative (•OH) or superoxidative (•O_2_^−^) species. Pollutant molecules subsequently adsorb and diffuse into the catalyst pores (step 4, Figure 3), where they react with highly reactive radical species and form compounds with lower molecular weights (H_2_O, CO_2_ and other intermediate products) [33,34,35].

The generally accepted reaction mechanism for organic pollutant photodegradation involves a radical pathway [36]. However, the direct oxidation of adsorbed organic pollutants by photogenerated holes has been reported [37,38]. The photocatalytic chain reaction is initiated by the hydroxyl radical formation (•OH) from H_2_O (+2.27 V vs. SHE), along with superoxide anion radical formation (•O_2_^−^) from O_2_ (−0.28 V vs. SHE), with the involvement of photogenerated h^+^ and e^−^. Hydroperoxyl radicals (•OOH) and H_2_O_2_ can form due to subsequent reactions of •O_2_^−^ with h^+^. Total VOCs mineralization is the goal of the photocatalytic applications. Unfortunately, many studies indicate the occurrence of partial organic pollutant oxidation, which may induce the formation of other pollutant molecules. The optimization of photocatalyst materials and photocatalytic parameters is a pre-requisite to improve the VOCs degradation efficiency [39,40].

(1) $$H_{2}O+h_{VB}^{+}\to •OH+H$$

(2) $$O_{2}+e_{CB}^{-}\to •O_{2}^{-}$$

(3) $$•O_{2}^{-}+H^{+}\to •OOH$$

(4) $$•OOH+•OOH\to H_{2}O_{2}+O_{2}$$

(5) $$H_{2}O_{2}+e_{CB}^{-}\to •OH+HO^{-}$$

## 3. VOC Removal Using Heterostructures/Composites/Doped Photocatalysts

Several VOCs with negative impacts on human health have been considered for this mini-review. Table 1 contains the key parameters presented in the scientific literature that may influence the photocatalytic activity of the samples. However, there are many other papers and parameters which are not presented here but can have an important impact in this field. Modified photocatalysts have the advantage of enhanced photocatalytic activity based on the insertion of dopant ions or by coupling with other materials. Most of these changes involve the optimization of intrinsic properties based on the energetic levels of the photocatalysts.

### 3.1. Toluene Photocatalytic Removal

Toluene is considered as an important VOC pollutant with major health hazard implications for the human body. Some of the short-term exposure symptoms to toluene are eye and nose irritation, weakness, exhaustion, confusion, etc. [41,42]. Long-term exposure to toluene induces central nervous system, respiratory system, liver and kidney damage [43]. Using a 300 W Vis light source, two heterostructures developed by in situ growth (TiO_2_-MIL-101(Cr) [44]) and hydrothermal (CoO/WO_3_ [45]) methods were employed for photocatalytic removal of toluene. TiO_2_-MIL-101(Cr) with an octahedral particle morphology and a high specific surface (2128 m^2^/g) was tested in 1000 ppm toluene for a 480 min irradiation period in order to achieve 50% photocatalytic efficiency. A CoO/WO_3_ p-n heterostructure with a plate-shaped morphology was used as a photocatalyst in 500 ppm toluene and the irradiation period was 240 min. The energy consumption is lower for CoO/WO_3_ using 1200 Wh to remove 427 ppm toluene due to CoO’s ability to increase the heterostructure Vis light absorbance. The p-type CoO has a negative enough conduction band (CB) to produce more active species, like superoxide radicals. Therefore, it is recommended that the p-type band position of CoO is well matched with that of n-type WO_3_. In this way, it is possible to efficiently suppress the recombination of photogenerated charge carriers, thereby enhancing the photocatalytic performance. Using a TiO_2_-MIL-101(Cr) heterostructure, it was possible to remove 500 ppm toluene but with the expense of 2400 Wh. The 50% photocatalytic efficiency was obtained based on the synergy effect between two heterojunctions (type II and surface heterojunctions) at the interface contact region of the semiconductors.

**Figure 4:**
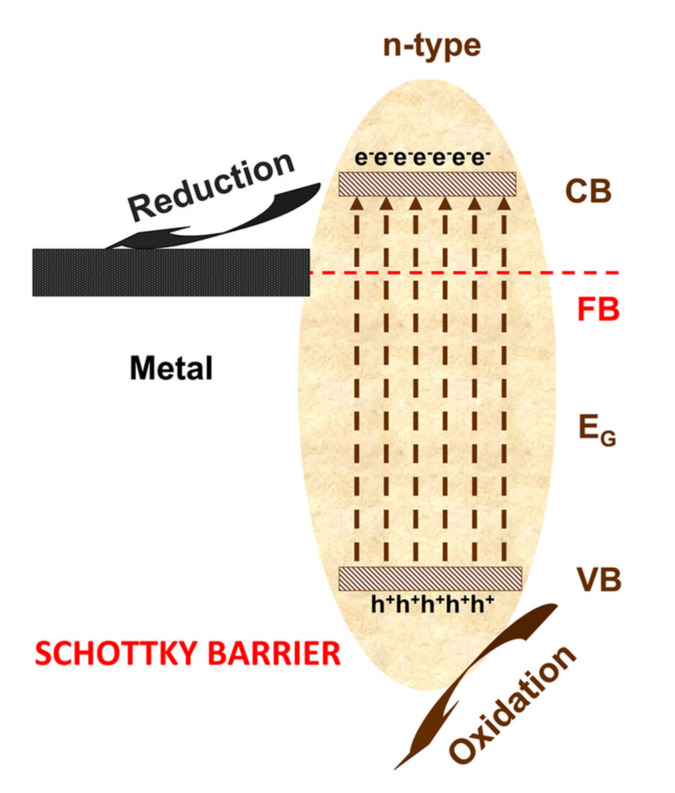
Schottky junction mechanism.

Keeping the same intensity (300 W) but changing the light spectra to the UV region, the CaCO_3_/TiO_2_ [46] photocatalytic activity was evaluated at 50 ppm toluene. A CaCO_3_/TiO_2_ heterostructure with an irregular particle morphology obtained by the dip-coating method have the advantage of improved gas molecule adsorption due to the CaCO_3_ surface chemistry. After 60 min of irradiation, the photocatalytic efficiency was 90%, which represents a 45 ppm toluene reduction with an energy consumption of 300 Wh. By increasing the UV light intensity up to 500 W, the Ag/TiO_2_ [47] Schottky junction heterostructure (see Figure 4) exhibits 99.6% photocatalytic efficiency toward 2.6 ppm toluene after a 180 min irradiation period. The Schottky junction develops at the semiconductor-metal interface and is able to reduce charge carrier recombinations and to promote the increase in semiconductor spectral light absorption. A significant feature of the Schottky junction is represented by the localized surface plasmonic resonance (LSPR) effect. Based on the LSPR, the photocatalysts have good visible light absorption and a significant excitation of active electron/hole pairs. The LSPR is present when the metal work function surpasses that of the semiconductor, inducing a positive space-charge region on the photocatalyst surface as well as upward semiconductor bands. The energy consumption is relatively high, considering that 1500 Wh were required to remove 2.59 ppm toluene. A Ag/TiO_2_ heterostructure with a nanotube morphology obtained by photoreduction was also employed to remove 3.3 ppm *N,N*-dimethylformamide (92.5% photocatalytic efficiency), 4.2 ppm acetone (99.4%) and 1.7 ppm dimethyl fumarate (99.7%). In all cases, the photocatalytic efficiency was high due to the nanotube structures allowing a facile diffusion of gaseous molecules and exposure to active sites. The Schottky junction based on the Ag nanoparticles’ uniform distribution inside the bottom-opening titania nanotubes contribute additional charge carriers required to obtain the oxidative species. 

A comparative study regarding the influence of light spectra on the 45.5 ppm toluene removal was done using a TiO_2_@MgAl [48] heterostructure with a 94.71 m^2^/g specific surface. A TiO_2_@MgAl heterostructure with a plate-like morphology was obtained by the in situ hydrolysis method. The photocatalytic activity was tested in the presence of a 500 W simulated sunlight source and in real sunlight during a 180 min irradiation period. In both situations, the photocatalytic efficiency was relatively high (91.7% in real sunlight and 85.9% in simulated sunlight), with an energy consumption of 1500 Wh to remove 39 ppm toluene (simulated sunlight). The photocatalytic activity was enhanced by the large plate surface area and the ionization ability of metals ions in the presence of water molecules. Another comparative study was done on a WO_3_/TiO_2_ type II heterostructure [49] by changing the top and bottom layers (WO_3_/TiO_2_ and TiO_2_/WO_3_) or by applying a bias (0.2 V). The heterostructures were obtained by the screen printing technique to make films with a 20 μm thickness. The photocatalytic experiments were done in 250 ppm toluene under 5 mW/cm^2^ irradiance for a 30 min period. The results indicate a 2.85 times higher photocatalytic activity for WO_3_/TiO_2_ (40%) compared to TiO_2_/WO_3_ (14%) and almost double the photocatalytic efficiency (70% for WO_3_/TiO_2_) when applying a 0.2 V bias. In the presence of an external bias voltage, the photogenerated electrons can be drawn away via the external circuit, leaving the photogenerated holes for toluene mineralization. For the WO_3_/TiO_2_ heterostructure, the photogenerated electrons in the CB of TiO_2_ could migrate to the CB of WO_3_. The electrons could be easily orientated to the external circuit through the WO_3_ layer at a low voltage, which acts as an unimpeded conduction passageway for charge carrier migration.

### 3.2. Photocatalytic Removal of Formaldehyde and O-xylene

Low concentrations of formaldehyde in air (0.1 ppm) produce burning sensations in the eyes and throat, nausea and skin irritation [50,51]. Long-term exposure to formaldehyde may cause severe diseases such as leukemia or cancer of the nasal sinuses [52,53]. The photocatalytic removal of formaldehyde was tested under Vis irradiation using graphene oxide GO/MnO_x_/Carbon Nanotubes (CNs) [54] and CeO_2_@layered double hydroxide (LBH) [55] heterostructures. GO/MnO_x_/CNs exhibit a sheet-like morphology with a bumpy surface structure and enhanced light absorption due to the scattering effect. The heterostructure reaches 90% photocatalytic efficiency for 160 ppm formaldehyde after 12 min of irradiation with a 300 W Vis light source. The GO/MnO_x_/CN heterostructures benefit from the improved synergetic photothermocatalysis and photocatalysis effects on decomposing HCHO. The mechanism rapidly enhances the surface temperature of photocatalysts under Vis irradiation and then drives the thermocatalysis of MnO_x_. The use of 2D/2D/2D nanosheet assembly increases the charge carrier transfer mechanism and well as the mobility between the sheet-like structures. Based on the temperature variation, the oxygen atom lattice can be activated in order to promote the transfer of photogenerated charge carriers to the carbon nitrate surface and the subsequent surface reactions. The results indicate an optimum energy consumption of 60 Wh to remove 144 ppm formaldehyde. Higher energy consumption was reported for CeO_2_@LBHs, which require 2500 Wh to remove 22.6 ppm formaldehyde. CeO_2_@LBHs were obtained by the hydrothermal method and have a petal-like lamella morphology, inducing a large specific surface (93 m^2^/g). The heterostructure follows a Z-scheme heterojunction mechanism where the photogenerated electrons on the conduction band of CeO_2_ are transferred to the valence band of CoAl-LDHs.

The main effect of inhaling o-xylene is depression of the central nervous system, with symptoms such as dizziness, headache or vomiting [56,57]. Chronic exposure may lead to insomnia, tremors, short-term memory loss and impaired concentration [58,59]. Reduced graphene oxide rGO-TiO_2_ [60] with a sheet-like morphology was obtained by the solvothermal method and contains TiO_2_ particles with 8–10 nm diameters. The photocatalytic activity was evaluated toward 25 ppm o-xylene using a 200 W Vis light source and a 160 min irradiation period. The results indicate a 54% photocatalytic efficiency at 533.3 Wh of energy consumption. A similar experiment involving rGO-TiO_2_ [60] was done with 25 ppm acetaldehyde and the photocatalytic efficiency was 42%. In this case, with the same energy consumption (533.3 Wh), the quantity of pollutant removal was lower (10.5 ppm reduction). When rGO is combined with titania, the specific surface area (227.3 m^2^/g) and the π–π bonds in graphene improve the organic compound adsorption. The decrease in intermolecular potential energy, which facilitates the adsorption process, is a consequence of dipole-dipole interactions between the rGO and the pollutant. The π-electron-rich active sites of rGO present a high affinity toward o-xylene molecules, which explains the enhanced photocatalytic activity from the initial stage. In the composite material, some of the electrons were trapped by the rGO sheet defects, prolonging the lifetime of the holes.

### 3.3. Photocatalytic Removal of Benzene and Ethylbenzene

Long-term exposure to benzene (and related compounds such as ethylbenzene, phenol, benzaldehyde, etc.) has been associated with a wide range of chronic health effects, including aplastic anemia (bone marrow is no longer able to produce enough red blood cells) and various forms of cancer [61,62]. Additionally, it can damage the immune system due to changes in the level of antibodies in the blood [63]. UV light with a 300 W intensity (TiO_2_/porous cementitious (PC) material [64]) and Vis light with a 100 W intensity (Cu-NiWO_4_ [65]) were employed to evaluate the photocatalytic removal of benzene. TiO_2_/PC was obtained by the negative pressure co-stirring method and exhibited a porous morphology with a 26 m^2^/g specific surface. The photocatalytic activity was evaluated for the removal of 200 ppm benzene during 180 min of UV irradiation. Low-density PC materials are useful to disperse the loaded titania particles due to the loose network of pores of needle-like hydrates, while the high-density substrates induce the agglomeration of loaded TiO_2_ particles. The photocatalytic efficiency was 63%, which represents 900 Wh of energy consumption to remove 126 ppm benzene. A Cu-NiWO_4_ heterostructure uses 4.5 times less energy (200 Wh) to remove 2.6 times less benzene (48.25 ppm). The presence of a Cu dopant induces the formation of medium-band energy between the conduction band and valence band of NiWO_4_, effectively reducing the electron–hole recombination rate. Based on the Cu dopant concentration, the photogenerated charge carriers have enough reduction or oxidation potential to react with oxygen and water molecules to form oxidative species. In this case, the high photocatalytic efficiency (96.5%) under 100 W Vis light source irradiation for 120 min was attributed to a high concentration of oxygen defects, inducing an increase in the benzene adsorption.

**Figure 5:**
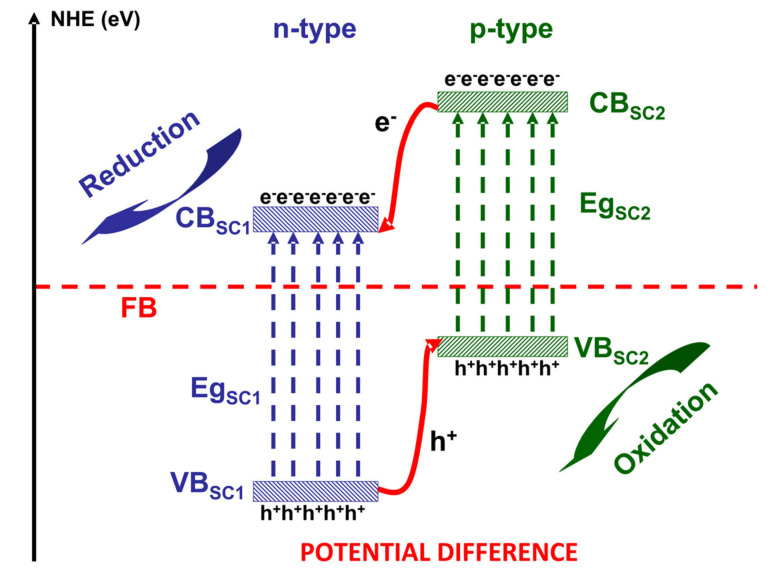
Type II heterostructure mechanism.

A BiVO_4_/TiO_2_ type II heterostructure [66] was employed for the evaluation of the influence of light spectra on the 260 ppm removal of benzene. Type II heterostructures (see Figure 5) are characterized by a potential difference formed between two semiconductors which separate the photogenerated charge carriers and prevent recombination processes. The reason is that the energy bands of one semiconductor have higher values compared to those of the second semiconductor. In this way, there is an effective separation between the holes and electrons on both sides.

A heterostructure with irregular particles was obtained by the hydrothermal method and was irradiated with 500 W solar simulated light, Vis and UV sources for 480 min. For the same energy consumption (~4000 Wh), the benzene removal in solar simulated light (240 ppm reduction) and Vis light (173.6 ppm reduction) was significantly higher compared to UV light (28.6 ppm reduction) irradiation. The photocatalytic activity uses BiVO_4_ as a sensitizer for the visible light-induced redox process, while TiO_2_ represents the substrate. Because BiVO_4_ is considered as an intrinsic semiconductor, its Fermi level lies in the middle of the conduction band and valence band.

Low-intensity UV radiation sources were used to determine the photocatalytic properties of SnO_x_/Zn_2_SnO_4_ [67] and Fe-TiO_2_ [68] samples. A SnO_x_/Zn_2_SnO_4_ heterostructure with rods (5 nm diameter and 100 nm length) and flake-like particles and a 21.7 m^2^/g specific surface was obtained by the hydrothermal method. After 840 min of irradiation with a 9 W UV source, the photocatalytic efficiency reached 80.3%. The benzene concentration was reduced to 200 ppm from 250 ppm using only 126 Wh of energy. SnO_x_/Zn_2_SnO_4_ follow a type II heterojunction mechanism, where the photogenerated electrons transfer from SnO_2_ to Zn_2_SnO_4_ due to the energy offset, while the photogenerated holes migrate from the valence band (VB) of Zn_2_SnO_4_ to the VB of SnO_2_. A similar study was done using a 8 W UV source to irradiate Fe-TiO_2_ nanoparticles for only 5 min. The authors [68] used a low benzene concentration (0.1 ppm), and the photocatalytic efficiency was 33%, which corresponds to the removal of 0.033 ppm with 0.66 Wh of energy consumption. Fe-TiO_2_ photocatalytic activity was also evaluated for toluene, ethylbenzene and o-xylene using the same VOC concentration and setup parameters. The photocatalytic efficiency varied from 68% for toluene to 83% for ethylbenzene and 91% for o-xylene due to the surface absorbance specificity for certain molecules. Additionally, Fe^3+^ species could act as electron and hole traps, reducing charge carrier recombination and improving photocatalytic performance. The excess doping of TiO_2_ with transition metals could increase the number of electron-hole recombination centers or even completely block the reaction sites. This process will drastically reduce the photocatalytic activity and this can be prevented by controlling the amount of transition metals related to the photocatalyst crystalline structure and composition. Higher photocatalytic efficiency (99%) for 11.5 ppm ethylbenzene was obtained by UV irradiation of La-TiO_2_ [69] for 1 min. La-TiO_2_ was obtained by combining the sol-gel and hydrothermal methods. The significant improvement of the photocatalytic efficiency corresponding to 1.2% La^3+^ doping may be attributed to the tubular structure and large surface area of nanotubes (541.35 m^2^/g), inducing homogenous reactant adsorption.

### 3.4. Acetaldehyde Photocatalytic Removal

Acetaldehyde is usually considered as an intermediate in the chemical synthesis of different products. Short-term exposure induces symptoms including irritation of the skin, respiratory tract and eyes [70,71]. Long-term exposure may be responsible for laryngeal or nasal cancerous tumors [72,73]. The photocatalytic activity of rGO-TiO_2_ [74] and TiO_2_/TaS_2_ [75] was evaluated under a 260 W Vis light source using 500 ppm acetaldehyde. The rGO-TiO_2_ with a sheet-like morphology was obtained by the ultrasonication method. After 60 min of irradiation, the photocatalytic efficiency was 80%, corresponding to a 400 ppm reduction with 260 Wh of energy consumption. Compared with rGO-TiO_2_, TiO_2_/TaS_2_ has a similar morphology and was obtained using the same synthesis method (ultrasonication). However, TiO_2_/TaS_2_ exhibited a higher photocatalytic efficiency (98%) due to a longer exposure period (65 min vs. 60 min) and a higher specific surface (103 m^2^/g vs. 69.81 m^2^/g). The energy consumption for a 490 ppm acetaldehyde reduction was 281.6 Wh. TaS_2_ provided two merits: (i) the increase in adsorptive capacity (three times bigger than that of TiO_2_) and (ii) higher photo-current (four times bigger than that of TiO_2_). TaS_2_ is considered as a typical two-dimensional material that can be integrated in composite structures or used as contact layer between two semiconductors. An advantage of using TaS_2_ is represented by its large specific surface area and low electrical resistivity which are beneficial in gaseous photocatalysis. Additionally, TaS_2_ increases the separation efficiency of photogenerated charge carriers.

Keeping the same radiation intensity (260 W) but switching from Vis to UV, the photocatalytic activity of Ag@TiO_2_ [76] and carbon quantum dots (CQDs)/TiO_2_ [77] was evaluated. Ag@TiO_2_ with a wire-like morphology and a 105.9 m^2^/g specific surface was obtained by the solvothermal method. After 4.8 min of irradiation, the sample exhibited 72% photocatalytic efficiency, which represented only 20.8 Wh of energy consumption to remove 360 ppm from 500 ppm acetaldehyde. The excellent photocatalytic activity is based on the Schottky junction, which increases the charge carrier concentration and mobility through the structure. CQDs/TiO_2_ with an irregular morphology were obtained by ultrasonication and the photocatalytic tests were done in the same conditions as for Ag@TiO_2_ but the irradiation period was 120 min. The increase in photocatalytic efficiency, up to 99%, was detrimental to the energy consumption, which was bigger (520 Wh) compared to that for Ag@TiO_2_. CQDs could serve as electron reservoirs by harvesting and retaining the photogenerated electrons from the conduction band of TiO_2_. During UV irradiation, the trapped photogenerated electrons on CQDs could further reduce the absorbed O_2_ to superoxidative radicals (•O_2_^−^). If the same sample is irradiated with a 400 W Vis light source for 120 min, the photocatalytic efficiency decreases significantly (30%) due to lower TiO_2_ photoconversion.

Highly concentrated acetaldehyde (3000 ppm) was used to evaluate the photocatalytic activity of a SiO_2_/TiO_2_ [78] type II heterojunction with a 20.5 m^2^/g specific surface obtained by the fiber impregnation method. The evaluation was done with and without plasma assistance and the results showed that total pollutant degradation is possible by using 22.6 W UV light source irradiation. The irradiation period is significantly reduced with plasma assistance (90 min) compared to without plasma assistance (140 min). These methods are difficult to compare in terms of energy consumption because the energy saved from the reduced irradiation period is used for plasma production. The reasons for coupling the photocatalysis process with plasma is the fact that plasma treatment interferes with oxidation reactions during the pollutant degradation and generates UV radiation (due to excited nitrogen relaxation), which is beneficial for TiO_2_ anatase photocatalytic activity. Additionally, the photocatalysis process can have an important influence on the improvement of carbon dioxide selectivity and mineralization balance.

A double CuInS_2_/TiO_2_/SnO_2_ [79] type II and p-n heterojunction was employed to evaluate the photocatalytic properties under UV-Vis irradiation. CuInS_2_/TiO_2_/SnO_2_ with a porous morphology (25.3 m^2^/g) was obtained by spray pyrolysis deposition. After 720 min of irradiation using a 20 W UV-Vis light source, the photocatalytic efficiency reached 51.7%. CuInS_2_/TiO_2_/SnO_2_ benefits from the synergic effect of dual type II and p-n heterojunctions, which allow for simultaneous charge photogeneration at each heterostructure component and lower recombination rates. Consequently, the energy consumption is 240 Wh in order to remove 258.5 ppm acetaldehyde. The photocatalytic efficiency depends on the intimate contact at the interface between components, as well as the surface chemistry in relation to the pollutant molecules. The degradation mechanism is positively influenced by the simultaneous charge carrier photogeneration of the heterostructure components and the increased mobility due to the suitable position of the energy bands.

### 3.5. Photocatalytic Removal of 2-ethyl-1-hexanol, N-decane, N-hexane, Trichloroethylene and Benzaldehyde

The photocatalytic removal of 2-ethyl-1-hexanol [80] and n-decane [81] was evaluated under Vis irradiation for 300 min. GO/TiO_2_ [80] with a sheet-like morphology and a large specific surface (100.3 m^2^/g) was obtained by ultrasonication and tested for the removal of low-concentration (0.1 ppm) 2-ethyl-1-hexanol. GOs in GO/TiO_2_ under visible light illumination act as sensitizers (electron donors) of titania that transfer the photogenerated electrons from the conduction band of GO/TiO_2_, reducing the recombination of photogenerated charge carriers and enabling GO/TiO_2_ to work under visible light irradiation. The 99.3% photocatalytic efficiency was obtained with low energy consumption (40 Wh) after 300 min of irradiation with a 8 W Vis light source, however, the initial pollutant concentration was only 0.1 ppm. Cellulose acetate (CA)/TiO_2_-P25 [81] with a porous morphology, obtained by the cold spray method, was irradiated with a 1700 W Vis light source to evaluate the photocatalytic removal of 320 ppm n-decane. The experimental investigations indicated that the photocatalytic reaction rate depends on the water/n-decane molar ratio. The competitive adsorption of water on the catalyst surface is favored by high water/n-decane ratios and reduces the adsorption of n-decane molecules, resulting in lower reaction rates. The photocatalytic efficiency was 72% and corresponded to a high energy consumption (8500 Wh for 230.4 ppm reduction). 

The accumulation of n-decane and n-hexane indoors from paint and solvents products can induce an increased number of conjuctival polymorphonuclear leukocytes and irritation of the mucous membranes of the eyes [82,83,84]. Bi/BiOBr [85] with a spherical particle morphology, obtained by the solvothermal method, was employed to evaluate 15 ppm n-hexane removal using a 300 W Vis light source. The Schottky Bi/BiOBr junction greatly inhibits the photogenerated carrier recombination rate, thereby promoting the accumulation of photoinduced electrons and holes in the conduction and valence bands of BiOBr to produce highly concentrated reactive oxygen species. A synthesis method that allows for the simultaneous formation of metallic Bi and BiOBr has the advantage of maintaining the BiOBr structure and imitates the chemical reaction occurring between Bi and BiOBr. The band gap narrowing for improved visible light absorption occurs due to the chemical bonding between Bi and BiOBr. Consequently, the photogenerated charge carriers increase, as well as the Bi/BiOBr composite photocatalytic activity. The photogenerated electron migration occurs from the conduction band of BiOBr to metallic Bi, improving the negative charge accumulation on Bi. This is the pathway to improve the photogenerated charge carrier separation on BiOBr, resulting in a higher charge lifetime. After 120 min of irradiation, the Bi/BiOBr sample exhibited 97.4% photocatalytic efficiency which represented 600 Wh of energy consumption to remove 14.6 ppm n-hexane.

The UV photocatalytic removal of trichloroethylene [86] and benzaldehyde [87] was evaluated with different photocatalytic materials. TiO_2_/SiO_2_ [86] with a granular morphology and a large specific surface (300 m^2^/g) was obtained by the dip-coating method. The photocatalytic activity was evaluated with 26 ppm trichloroethylene for a 240 min irradiation period. The total trichloroethylene removal was a consequence of higher gas affinity on the surface of the silica-based material. SiO_2_ particles, with their high surface area, represent a buffer and provide a suitable medium for trichloroethylene accumulation, resulting in higher overall conversion. TiO_2_/conductive carbon felt (OMT/CCF) [86] with a fiber-like morphology (148.6 m^2^/g specific surface) was obtained by the liquid crystal template method with the assistance of ultrasonic deposition. The photocatalytic removal of 100 ppm benzaldehyde at 40 mW/cm^2^ irradiance was tested for a 325 min irradiation period and the photocatalytic efficiency was ~25% due to the synergistic relationship between CCF, which acts as an excellent electron acceptor and organic gas concentrator, and OMT, representing a reactant transporter and reactor, thus reducing the photogenerated charge carrier recombination.

**Table 1:** Representative studies on photocatalytic applications for volatile organic compound (VOC) removal.

Heterostructure/ Composite/ Doped Photocatalysts	Synthesis Method	Specific Surface (S_BET_)/Radiation Parameter (Light Spectra, Intensity and Irradiance)/Energy Consumption (E_C_)	Pollutant/Photocatalytic Parameters	Ref.
TiO_2_-MIL-101 (Cr)	In situ growth on MIL101 (Cr)	S_BET_ = 2128 m^2^/ g300W Vis, np* E_c_ = 2400 Wh	Toluene Pollutant concentration: 1000 ppm Time: 480 min Efficiency: ~50% Rate constant: np	[44]
CoO/WO_3_	Hydrothermal	S_BET_ = np 300W Vis, np E_c_ = 1200 Wh	Toluene Pollutant concentration:500 ppmTime: 240 min Efficiency: 85.4% Rate constant: 0.0070 min^−1^	[45]
CaCO_3_ loading TiO_2_	Dip coating	S_BET_ = np 300W UV, 0.29 W/cm^2^ E_c_ = 300 Wh	Toluene Pollutant concentration: 50 ppm Time: 60 min Efficiency: 90% Rate constant: np	[46]
Ag/TiO_2_	Photoreduction method	S_BET_ = np 500 W UV, np E_c_ = 1500 Wh	Toluene, N,N-dimethylformamide, acetone, dimethyl fumaratePollutant concentration: 2.6 ppm (toluene), 3.3 ppm (N,N-dimethylformamide), 4.2 ppm (acetone), 1.7 ppm (dimethyl fumarate) Time: 180 min Efficiency: 99.6% (toluene), 92.5% (N,N-dimethylformamide), 99.4% (acetone), 99.7% (dimethyl fumarate) Rate constant: np	[47]
TiO_2_@MgAl-layered double hydroxide	In situ hydrolysis	S_BET_ = 94.71 m^2^/ g500 W Vis, np Real sunlight, np E_c_ = 1500 Wh	ToluenePollutant concentration: 45.5 ppm Time: 180 min Efficiency: 91.7% (true sunlight), 85.9% (simulated sunlight) Rate constant: 0.0100 min^−1^	[48]
WO_3_/TiO_2_	Screen printing technique	S_BET_ = np UV, 5 mW/cm^2^ E_c_ = np	Toluene Pollutant concentration: 250 ppm Time: 30 min Efficiency: 14% (T/W), 40% (W/T), 70% (W/T with 0.2 V bias) Rate constant: 0.00739 min^−1^ (T/W), 0.02004 min^−1^ (W/T)	[49]
Graphene oxide (GO)/MnO_x_/CN	Vacuum filtration	S_BET_ = np 300 W Vis, np E_c_ = 60 Wh	Formaldehyde Pollutant concentration: 160 ppm Time: 12 min Efficiency:90% Rate constant: 0.202 min^−1^	[54]
CeO_2_@ layered double hydroxides	Hydrothermal	S_BET_ = 93 m^2^/ g500 W Vis, np E_c_ = 2500 Wh	Formaldehyde Pollutant concentration: 26 ppm Time: 300 min Efficiency: 86.9% Rate constant: 0.00101 min^−1^	[55]
Reduced graphene oxide (rGO)-TiO_2_	Solvothermal	S_BET_ = 227.3 m^2^/g 200W Vis, np E_c_ = 533 Wh	Acetaldehyde, o-xylene Pollutant concentration: 25 ppm Time: 160 min Efficiency: 42% (acetaldehyde), 54% (o-xylene). Rate constant: np	[60]
TiO_2_/porous cementitious material	Negative pressure co- stirring method	S_BET_ = 26 m^2^/g 300W UV, 0.96 mW/cm^2^ E_c_ = 900 Wh	Benzene Pollutant concentration: 200 ppm Time: 180 min Efficiency: 63% Rate constant: np	[64]
Cu-NiWO_4_	Sol-gel	S_BET_ = 12.4 m^2^/g 100W Vis, 0.025 W/cm^2^E_c_ = 200 Wh	Benzene Pollutant concentration: 50 ppm Time: 120 min Efficiency: 96.5% Rate constant: np	[65]
BiVO_4_/TiO_2_	Hydrothermal	S_BET_ = 66.49 m^2^/g 500 W solar simulated light, np 500 W Vis, np 500 W UV, np E_c_ = 4000 Wh	Benzene Pollutant concentration: 260 ppm Time: 480 min Efficiency: 92% (solar simulated light), 66.8% (Vis), 11% (UV) Rate constant: np	[66]
SnO_x_/Zn_2_SnO_4_	Hydrothermal	S_BET_ = 21.7 m^2^/g 9 W UV, np E_c_ = 126 Wh	Benzene Pollutant concentration: 250 ppm Time: 840 min Efficiency: 80.3% Rate constant: 0.0834 min^−1^	[67]
Fe-TiO_2_	Electrospinning technique	S_BET_ = np8W UV, 0.4 mW/cm^2^ E_c_ = 0.66 Wh	Benzene, toluene, ethylbenzene and o-xylene Pollutant concentration: 0.1 ppm Time: 5 min Efficiency: 33% (benzene), 68% (toluene), 83% (ethylbenzene) and 91% (o-xylene) Rate constant: np	[68]
La-TiO_2_	Sol–gel method and hydrothermal technique	S_BET_ = 541.35 m^2^/g UV, 20.9 mW/cm^2^ E_c_ = np	Ethylbenzene Pollutant concentration: 11.5 ppm Catalyst dosage: 1 min Efficiency: 99% Rate constant: 1.1860 min^− 1^	[69]
Reducend graphene oxide (rGO) with TiO_2_	Ultrasonication	S_BET_ = 69.81 m^2^/g 260 W Vis, np E_c_ = 260 Wh	Acetaldehyde Pollutant concentration: 500 ppm Time: 60 min Efficiency: 80% Rate constant: np	[74]
TiO_2_/TaS_2_	Ultrasonication	S_BET_ = 103 m^2^/g 260 W Vis, np E_c_ = 281 Wh	Acetaldehyde Pollutant concentration: 500 ppm Time: 65 min Efficiency: 98% Rate constant: 0.03091 min^−1^	[75]
Ag@TiO_2_	Solvothermal	S_BET_ = 105.93 m^2^/g 260W UV, 20 mW/cm^2^ E_c_ = 20.8 Wh	Acetaldehyde Pollutant concentration: 500 ppm Time: 4.8 min Efficiency: 72% Rate constant: 0.01199 min^−1^	[76]
Carbon quantum dots/TiO_2_	Ultrasonication	S_BET_ = np 260W UV, 20 mW/cm^2^ 400W Vis, np E_c_ = 520 Wh (UV) E_c_ = 800 Wh (Vis)	Acetaldehyde Pollutant concentration: 500 ppm Time: 120 min Efficiency: 99% (UV), 30% (Vis) Rate constant: np	[77]
SiO_2_/TiO_2_	Fiber impregnation	S_BET_ = 20.5 m^2^/g 22.6 W UV, 6 mW/cm^2^ E_c_ = 52.7 Wh (photocat) E_c_ = 34 Wh (plasma photocat)	Acetylene Pollutant concentration: 3000 ppm Time: 140min (photocat), 90 min (plasma photocat) Efficiency: 100% Rate constant: np	[78]
CuInS_2_/TiO_2_/SnO_2_	Spray pyrolysis deposition	S_BET_ = 25.3 m^2^/g 20 W UV + Vis, (2.5 mW/cm^2^ +0.1 mW/cm^2^) E_c_ = 240 Wh	Acetaldehyde Pollutant concentration: 500 ppm Time: 720 min Efficiency: 51.7% Rate constant: 0.0557 min^−1^.	[79]
GO/TiO_2_	Ultrasonication	S_BET_ = 100.3 m^2^/g 8 W Vis, np E_c_ = 40 Wh	2-ethyl-1-hexanol Radiation: Vis Pollutant concentration: 0.1 ppm Time: 300 min Efficiency: 99.3% Rate constant: np	[80]
Cellulose acetate (CA)/TiO_2_-P25	Cold spray	S_BET_ = np 1700 W Vis, 38.4 W/m^2^ E_c_ = 8500 Wh	N-decane Pollutant concentration: 320 ppm Time: 300 min Efficiency: 72% Rate constant: np	[81]
Bi/BiOBr	Solvothermal	S_BET_ = np 300 W Vis, np E_c_ = 600 Wh	N-hexane Pollutant concentration: 15 ppm Time: 120 min Efficiency: 97.4% Rate constant: 0.0300 min^−1^	[85]
TiO_2_/SiO_2_	Dip coating	S_BET_ = 300 m^2^/g UV (254 nm and 365 nm) Ir_254_ = 7.3 × 10^−3^W/cm^2^ Ir_365_ = 3.5 × 10^−3^W/cm^2^ E_c_ = np	Trichloroethylene Pollutant concentration: 26 ppm Time: 240 min Efficiency: 100% Rate constant: np	[86]
Mesoporous TiO_2_/conductive carbon felt (OMT/CCF)	Liquid crystal template method with the assistance of ultrasonic deposition	S_BET_ = 148.6 m^2^/g UV, 40 mW/cm^2^ E_c_ = np	Benzaldehyde Pollutant concentration: 100 ppm Time: 325 min Efficiency: ~25% Rate constant: 0.0004 min^−1^	[87]

* not provided.

Finding new and innovative solutions to enhance the photocatalytic properties is a pre-requisite in consolidating AOPs as future alternatives for VOCs removal. It was found that doping TiO_2_ with Mn^2+^ or Co^2+^ [88] can increase the photocatalytic activity due to morphological changes, inducing a higher absorbance capability, and can shift the band gap toward the Vis range. Bismuth-doped titania [89], as well as eosin-modified TiO_2_ [90], were reported to have high photocatalytic activity due to intrinsic property changes, such as oxygen vacancy formation or light trapping. Titania, especially the anatase form, seems to be one of the most suitable photocatalysts, with high chemical stability and versatile morphologies. However, the main disadvantage is represented by the limited absorption in the UV range, which reduces the quantity of photons able to participate in the photocatalytic reactions. Processes like doping or coupling with other materials represent a suitable solution to improve the TiO_2_ absorption range and the overall photocatalytic efficiency. Based on the literature review, there is no specific photocatalyst that can be recommended for a certain pollutant molecule. Highly active photocatalysts can act indiscriminately on organic pollutants with several specifications: interface compatibility, concentration range and light spectral absorbance. There are specific ways to improve the overall photocatalytic efficiency, which depends on materials (monocomponent, multicomponent, doped, etc.) and process (mechanism, radiation type, intensity, etc.).

## 4. Conclusions

The real impact of VOCs in indoor air on human health is still a subject of research investigations considering the large increase in forms of cancer and related diseases. The implementation of modern materials and technologies in houses and workspaces has induced the increased concentration and diversification of VOCs. While waiting for health impact studies, prevention should be considered. Photocatalysis may represent a safe method to eliminate VOCs if complete pollutant mineralization occurs.

The photocatalytic efficiency for VOC removal depends not only on the photocatalyst composition but also on the morphology and specific surface. A sheet-like morphology seems to provide a larger number of active sites, which may contribute to the oxidative reactions. The insertion of materials able to increase light absorbance or to mediate the charge carrier transport will have a beneficial impact on the overall photocatalytic efficiency. Additionally, surface chemistry must be considered when developing photocatalysts for certain gas pollutants in order to favor molecule absorbance in the interfacial region.

Energy sustainability for future large-scale implementation requires significantly more experimental investigations. The scientific literature is abundant in various but rather non-uniform correlations regarding the energy demand for photocatalytic pollutant removal. There are studies which use high energy consumption to remove small quantities of pollutants or papers giving incomplete data on this subject. Standardization should be considered as a useful tool to compare and improve the research results in this field considering the high impact on human health. Nevertheless, the development of new photocatalytic materials or technologies, such as coupling photocatalysis with other techniques (adsorption, biodegradation, etc.), can be another pathway to follow for optimum energy consumption and organic pollutant removal.
